# Genetic Diversity and Population Structure Among Czech and Polish Goat Breeds Assessed Using Microsatellite Markers

**DOI:** 10.3390/ani16111660

**Published:** 2026-05-29

**Authors:** Zuzana Sztankóová, Emil Krupa, Emilia Bagnicka, Paulina Nazar, Monika Gregula Kania, Jana Rychtářová

**Affiliations:** 1 Department of Genetics and Breeding of Farm Animals, Institute of Animal Science, 104 00 Prague, Czech Republic; 2 Institute of Genetics and Animal Biotechnology, Polish Academy of Sciences, Postępu 36a, 05-552 Jastrzebiec, Poland; 3 Department of Animal Breeding and Agriculture Advisory, Faculty of Animal Sciences and Bioeconomy, University of Life Sciences in Lublin, Akademicka 13, 20-950 Lublin, Poland; 4 Department Biology of Reproduction, Institute of Animal Science, 104 00 Prague, Czech Republic

**Keywords:** goat, genetic diversity, microsatellite marker, population structure

## Abstract

Microsatellite markers were employed to determine the genetic diversity and structure of eight goat breeds kept in the Czech Republic (White Shorthaired goat, Brown Shorthaired goat, Alpine, Anglo-Nubian) and Poland (Polish White Improved, Polish Fawn Improved goat, Sandomierska, Toggenburg). Across 398 individuals analyzed using 15 microsatellite markers, a total of 191 alleles were detected, with a mean of 7.108 alleles per locus. The average of observed and expected heterozygosity, fixation index, and Shannon’s information index were 0.684 ± 0.014, 0.696 ± 0.013, 0.014 ± 0.012, and 1.476 ± 0.037, respectively. The mean of overall genetic differentiation (F_ST_) was 0.110, heterozygote deficiency among populations (F_IT_) was 0.125, and the inbreeding coefficient within the goat population (F_IS_) was 0.018. Analysis of molecular variance showed that 10% of the total genetic variation was attributed to differences among populations, 3% among individuals within populations, 86% within individuals, and 1% was observed among regions. A phylogenetic dendrogram and principal coordinate analysis (PCoA) revealed three main clusters, while STRUCTURE analysis identified two main gene pools. These findings provide critical insights for future genetic improvement, breeding program optimization, and conservation strategies for local landrace goat breeds, whose genetic resources are pivotal for maintaining livestock biodiversity.

## Simple Summary

Molecular characterization of local breeds is a key factor in biodiversity conservation. It should be combined with appropriate breeding strategies to improve the production characteristics of indigenous breeds and prevent breed decline. In this study, we analyzed 398 animals from eight goat breeds (White Shorthaired goat, Brown Shorthaired goat, Alpine, Anglo-Nubian, Polish White Improved goat, Polish Fawn Improved goat, Sandomierska, Toggenburg) bred in the Czech Republic and Poland using 15 microsatellite loci. All microsatellite markers were polymorphic. The Anglo-Nubian breed differed the most from the other observed breeds. The analysis revealed two main gene pools and showed a low level of inbreeding; the genetic markers used are suitable for genetic diversity analysis, and the obtained results can be used to develop effective strategies for their protection and conservation.

## 1. Introduction

Goats are among the first domesticated livestock species, dating back to 11,000 years ago in the Middle East region, and have played an important role in the development of human history. While their origin is polyphyletic, the primary ancestor of the domestic goat (*Capra hircus*) belongs to *Capra aegagrus* and *Capra falconeri* [1,2,3]. Based on the molecular study of mtDNA, goat breeds are classified into Haplogroups (A–G), whereas phenotypic evaluations categorize them into three groups [4,5,6].

There are around one billion goats in the world, distributed across 1234 serving breeds (https://www.fao.org/dad-is/en, accessed on 25 May 2026), and they are bred for their milk, meat, fiber, and skin. Goats are popularly known as the “poor man’s cow” because they provide a variety of benefits at a low cost. Along with sheep, goats are the most adaptable and geographically widespread livestock species, living in various ecosystems [4,7].

Nevertheless, there are numerous studies that have monitored the genetic diversity and population structure of local breeds for the purpose of either protecting local breeds as a genetic source, creating a breeding program to improve the production and functional traits of the local breed, or for the purpose of crossbreeding to increase production or for the purpose of creating a new breed. This creation leads to the displacement of native breeds into the background and their replacement by cultivated or hybrid breeds with the aim of increasing overall production (dairy or meat); however, genetic diversity is one of the components of biodiversity, such that it is necessary to control and manage inbreeding from the point of view of breed origin [6,7,8,9,10,11,12,13,14]. Indeed, intensive selection and the widespread replacement of native populations with highly productive cosmopolitan breeds have led to a severe loss of biodiversity and the marginalization of indigenous genetic resources [15]. The FAO further states that cryopreservation is one of the tools to protect and preserve populations from extinction or loss and to manage genetic diversity ex situ. Currently, there are many molecular techniques to study genetic variation, phenotype and pedigree information among livestock, including minisatellite and microsatellite markers and single-nucleotide polymorphism (SNP) [13,16,17]. Due to a high degree of polymorphism, codominant inheritance, and selection neutrality, reproducible microsatellite markers (MMs) are regarded as the most useful DNA markers [18]. However, there are other genotyping techniques for estimating genetic diversity in population studies, like whole-genome sequencing [19] and next-generation sequencing (NGS) [20].

The genetic diversity and structure of Czech breeds using MMs and at the level of genomic diversity have been described in the studies of Jandurova et al. [21] and Vostry et al. [22], respectively. However, in the period 1900–1960, the WSH breed was developed by crossing local breeds with Saanen goats imported from Switzerland and Germany, and the BSH breed was crossed with the German Harz goat, with both having been maintained as separate populations since 1929. The genetic structure of Polish autochthonous and cosmopolitan goat breeds via MMs, including Polish native Carpathian goat, Saanen, Alpine, and Anglo-Nubian, has been reported by Kawecka et al. [23,24] and Sikora et al. [25]. In the late 1980s, the Polish local breeds, especially in Lower Silesia, were improved using, among others, bucks of Czech White Shorthaired goats from the former Czechoslovakia; does were also imported. Since the Lower Silesian goat population was the largest in Poland at that time, it played a significant role in the reconstruction of the goat population in Poland, as a large part of the breeding material was distributed throughout the country, influencing the genotype of goats, especially of Polish White Improved Goats, described by Bagnicka et al. [26].

The aim of this study was to (i) compare and characterize the genetic diversity and population structure of national goat breeds (gene resources) with respect to geographical proximity, good resistance to climatic conditions, good productivity, and reproduction based on genotyping with microsatellite loci, in this case, using a set of 15 microsatellite markers recommended by FAO and (ii) evaluate the purity of indigenous goat breeds and compare the possible impact of genetic admixture between them. As described by Meyermans et al. [27], local breeds, whether sheep or goats, often play an important role in cultural heritage, can play a key role in local economies, have unique characteristics adapted to local climates, and can be invaluable in sustainable and resilient agricultural systems (maintenance of landscapes and biodiversity) (FAO).

## 2. Materials and Methods

### 2.1. Extraction of DNA

**Figure 1:**
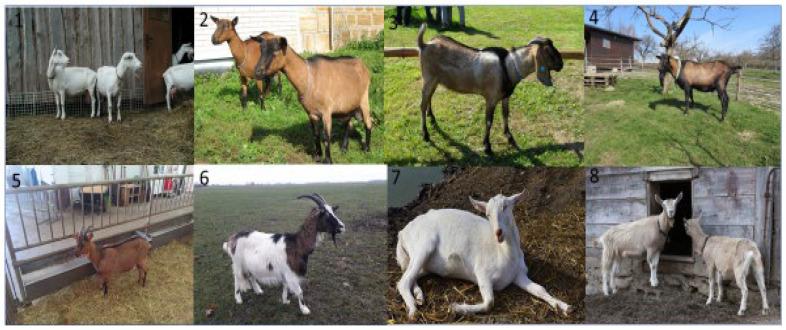
1: White Shorthaired goat, 2: Brown Shorthaired goat, 3: Anglo-Nubian, 4: Alpine, 5: Polish Fawn Improved goat, 6: Sandomierska goat, 7: Polish White Improved goat, 8: Toggenburger (Source: Czech population: VÚŽV, V. Mátlová, J. Pikousová, SCHOK, Polish population: E. Bagnicka, Z. Kołodziej, www.swiatrolnika.info, accessed on 25 May 2026).

Total DNA was isolated from 398 hair follicle samples from randomly selected unrelated individuals from multiple herds and national gene banks of 8 goat populations kept in the Czech Republic: White Shorthaired goat (WSH, 59), Brown Shorthaired goat (BSH, 59), Anglo-Nubian (ANG, 65), Alpine (ALP, 32), and kept in Poland: Polish Fawn Improved (PFI, 48), Sandomierska goat (SAND, 88), Polish White Improved (PWI, 31), Toggenburg (TOGN, 16) (Figure 1), using the NucleoSpin Tissue kit (Macherey-Nagel, Düren, Germany, IBiotech Ltd., Prague, Czech Republic) according to the manufacturer’s instructions. The quality and quantity of the DNA were checked on a 3% agarose gel and subsequently visualized on a UV transilluminator gel documentation system (G-box, Trigon Ltd., Čestlice, Czech Republic) after staining with ethidium bromide (EtBr) (Top-Bio Ltd., Vestec, Czech Republic). The isolated DNA was stored at −20 °C before analysis.

### 2.2. Microsatellite Markers

A panel of fifteen microsatellite markers—SRCRSP23, MAF65, MCM527, BM1329, ETH10, ILSTS11, TGLA 53, SPS113, SRCSP5, SRCSP8, INRA172, INRA006, INRA 63, INRA 23 and CSRD247—was used to genotyped eight goat breeds. All microsatellite markers were selected based on the guidelines of the International Society for Animal Genetics (ISAG) (https://www.Isag.org.uk/Docs/2005_panelsMarkersSheepGoats.Pdf, accessed on 25 May 2026).

### 2.3. PCR and Fragmentation Analysis

The 15 loci were divided into three multiplexes (Supplementary Table S1). Amplification was performed in a total reaction volume of 10 µL containing ~50 ng of genomic DNA, 5 µL of 2× MyTaq HS Mix (Bioline Ltd., London, UK; supplied by IBiotech Ltd., Prague, Czech Republic), 0.1–0.35 µL of fluorescently labeled primers (10 pmol/µL) and sterile water to the final volume. Amplification was performed in a thermal cycler (Biometra-TAdvance, IBiotech, Prague, Czech Republic) with an initial denaturation at 95 °C for 15 min, followed by 32 cycles of denaturation at 94 °C for 30 s, annealing at 49.5 °C for 90 s (for markers INRA06, INRA063 (D1855), INRA23, CSRD247) or at 55 °C for the remaining multiplexes (Panel 2: SRCRSP23, MAF65, MCM527, BM1329, ETH10, ILSTS11; Panel3: TGLA53-D1653, SPS113, SRCRSP5, SRCRSP8, INRABERN172), followed by extension at 72 °C for 1 min, and a final exposure at 72 °C was prolonged to 30 min.

Fragmentation analysis of the PCR product was performed by capillary electrophoresis using an automated ABI PRISM3130 genetic analyzer (Applied Biosystems, Foster City, CA, USA). Each injection mixture with a total volume of 11 μL contained 1 μL of the PCR products, 9.0 μL of Hi-Di formamide and 0.5 μL of GeneScan-500LIZ size standard, with 0.5 μL of redistilled water to make up the final volume. The mixture was denatured at 95 °C for 5 min (Biometra-TAdvance, IBiotech, Prague, Czech Republic) and immediately cooled on ice for 3 min. Allele sizing and genotyping results were determined by the GeneMapper V 4.1 program (Applied Biosystems, Foster City, CA, USA). Prior to analysis, rigorous genotype quality control (QC) was performed via visual inspection of electropherograms to verify allele calls and peak quality. Ambiguous samples were re-amplified or treated as missing data. Thresholds were set to exclude individuals with >20% missing data and flag loci with call rates <90%. Genotyping artifacts—such as null alleles, stutter peaks, and large allele dropout—were systematically evaluated. Potential null alleles were identified via heterozygote deficiencies but not excluded, consistent with comparable studies.

### 2.4. Data Analysis

Genetic diversity parameters were calculated using GenAlEx v. 6.5.1 software [28], including the number of different alleles (Na), number of effective alleles (Ne), number of private alleles (PA), Shannon’s information index (I), observed (Ho) and expected (He) heterozygosity, number of effective migrants (Nm), fixation indices (F_IS_, F_IT_ and F_ST_), and the Hardy–Weinberg equilibrium (HWE) test across loci and breeds. PowerMarker software v. 3.0 [29] was used to estimate the polymorphism information content (PIC) according to Botstein et al. [30], as well as genetic distances DS according to Nei M. (1972) [31], Rogers J.S. (1972, DR) [32], and Reynolds et al. (1983) [33]. The T-REX: a web server (www.trex.uqam.ca/index.php?action=trex&menud=1&method=2, accessed on 25 May 2026) was used to construct a Neighbor-Joining (NJ) phylogenetic tree based on Neighbor-Joining method [34]. In addition, structural analysis using STRUCTURE software version 2.3.4 [35] was implemented to investigate breed differentiation from multilocus genotyping utilizing a Bayesian approach clustering method. Assignment clusters were made with a length of 100,000, followed by 150,000 Markov Chain Monte Carlo (MCMC) iterations after burn-in with 10 iterations for each K. The optimal number of clusters (K) was determined using the Evanno (delta K) method [36], Raj et al. [37], and Puechmaille statistics [38], and all were implemented via the STRUCTURE SELECTOR (https://lmme.ac.cn/StructureSelector/index.html, accessed on 18 March 2026) [39] web tool, interface integrated with CLUMPAK software (http://clumpak.tau.ac.il/, accessed on 18 March 2026) [40].

## 3. Results

### 3.1. Genetic Diversity of Microsatellite Markers

A total of 191 alleles (K) were identified across 15 microsatellite loci in 398 genotyped individuals from eight goat populations comprising Czech and Polish breeds. The total number of alleles per locus (K) ranged from four (ETH10) to 23 (MAF65, CSRD247), with a mean of 12.733. The mean number of alleles per population (Na) was 7.108, and the average number of effective alleles was 3.908 (Table 1). Five out of the 15 microsatellite loci showed a significant deviation from the Hardy–Weinberg equilibrium (HWE) (*p* < 0.001). The mean Polymorphism Information Content (PIC) was 0.744, confirming that the selected marker panel was highly informative. The most informative loci were SRCRSP23 (0.862) and MAF65 (0.844), whereas ETH10 was the least informative locus (0.461) due to its lower allelic richness. The level of genetic variability was further evaluated using heterozygosity parameters (Table 1) and Shannon’s information index (I) (Table 2). The expected heterozygosity (He) per locus ranged from 0.465 (ETH10) to 0.774 (MAF65), with an overall population mean of 0.696. Concurrently, the observed heterozygosity (Ho) ranged from 0.473 (ETH10) to 0.810 (INRA006), with an overall mean of 0.684. The total genetic diversity (Ht) estimate based on pooled allele frequencies was 0.783, indicating a high level of genetic diversity within the entire dataset.

The mean Shannon’s information index across all loci was 1.476, ranging from 0.783 (ETH10) to 1.838 (MAF65) (Table 2), further confirming the high genetic diversity within the studied populations. Due to the high polymorphism and informativeness of all analyzed microsatellite loci, this marker panel proved to be highly efficient for estimating genetic variance and population parameters in these breeds. Population stratification and the potential loss of genetic variability were assessed using Wright’s F-statistics and Nei’s G-statistics (Table 2). The average fixation index (F_IS_) was 0.018, which was consistent with the analogous G_IS_ value of 0.031. These low estimates indicate a negligible level or complete absence of inbreeding among individuals within the studied subpopulations. The coefficient of genetic differentiation among subpopulations, F_ST_, was 0.110, demonstrating moderate genetic differentiation between the breeds. This finding was fully supported by the G_ST_ analysis, which revealed that 10.0% of the total genetic diversity was partitioned among breeds (G_ST_ = 0.100), whereas the remaining 90.0% of the genetic variance was maintained within the populations. Finally, the global heterozygote deficit across the entire metapopulation, F_IT_, was estimated at 0.125 (12.5%), and the mean number of effective migrants, Nm, was 2.419.

### 3.2. Genetic Variability Among Czech and Polish Goat Breeds

The genetic diversity parameters analyzed across the fifteen microsatellite loci for the eight Czech and Polish goat populations are summarized in Table 3 and Supplementary Table S2. The highest average number of alleles per population, Na, was observed in the Polish Sandomierska goat (Na = 10.07), whereas the lowest allelic richness was recorded in the Anglo-Nubian breed (Na = 5.27). Similar trends were observed for the number of effective alleles, Ne. The lowest observed heterozygosity, Ho, was found in the Anglo-Nubian breed (0.599), while the highest was reported in the Polish Fawn Improved (PFI) breed (0.721). Regarding the expected heterozygosity, He, the lowest value was again observed in the Anglo-Nubian (0.576), whereas the highest genetic variability was detected in the Sandomierska breed (0.744). The fixation index (F), reflecting the inbreeding coefficient, ranged from −0.041 (Anglo-Nubian) to 0.073 (Sandomierska), with an overall mean of 0.011, indicating a low level of inbreeding within the individual subpopulations. The analysis of private alleles (PA) revealed that the Sandomierska goat possessed the highest number of unique alleles (PA = 11), whereas the lowest number of private alleles was found in the Alpine (ALP) and Polish White Improved (PWI) breeds, both displaying only two private alleles (PA = 2).

Analysis of Molecular Variance (AMOVA) was performed to partition the genetic variation within and among the studied populations and geographical regions (Table 4). The AMOVA analysis revealed that the vast majority of the total genetic variance (86.0%) occurred within individuals. Variation among populations accounted for 10.0% of the total diversity, followed by variation among individuals within populations (3.0%). The lowest proportion of variance was attributable to differences among geographical regions (1.0%). The F_ST_ value derived from the AMOVA was 0.109, which was almost identical to the G_ST_ value (0.100) and highly consistent with the overall F_ST_ estimate (0.110) obtained in the single-locus analysis. These results confirm that the genetic differentiation between the breeds explains a significant portion of the existing genetic diversity.

### 3.3. Genetic Differentiation, Distance and Phylogeny

To evaluate the degree of genetic differentiation, the pairwise differentiation coefficient (F_ST_) was calculated among all population pairs (Table 5). The most significant genetic divergence was observed between the ANG and TOGN breeds (F_ST_ = 0.132), followed closely by ALP and ANG (F_ST_ = 0.131). Conversely, the lowest differentiation was detected among the Polish goat breeds, specifically between PWI and PFI (F_ST_ = 0.012), and between PWI and SAND (F_ST_ = 0.015). Regarding the Czech populations, the WSH and BSH breeds exhibited the highest genetic proximity (F_ST_ = 0.029). Furthermore, low levels of differentiation were observed between the Czech cohorts (WSH, BSH) and the Polish populations (SAND, PWI, PFI). Consistent with these F_ST_ patterns, the highest historical gene flow (Nm) was recorded for the PWI/PFI pair (19.935), followed by SAND/PWI (16.919), SAND/WSH (12.476), and SAND/PFI (12.463). The lowest gene flow values were observed between the ANG/TOGN (1.649) and ANG/ALP (1.657) breeds. These findings are fully congruent with the estimated number of migrants, the principal coordinate analysis (PCoA), and the phylogenetic dendrogram.

Table 6 and Table 7 describe the genetic distances among the observed goat populations, which were quantified using Nei’s genetic distance (DS) and Rogers’ genetic distance (DR) [32], as well as determined by the genetic distance according to Reynolds (1983) [33]. According to the DS calculation, the smallest genetic distance was recorded for the Polish breeds PWI and PFI (0.076), whereas the most distant breeds were ALP and ANG (0.788) (Table 5).

**Figure 2:**
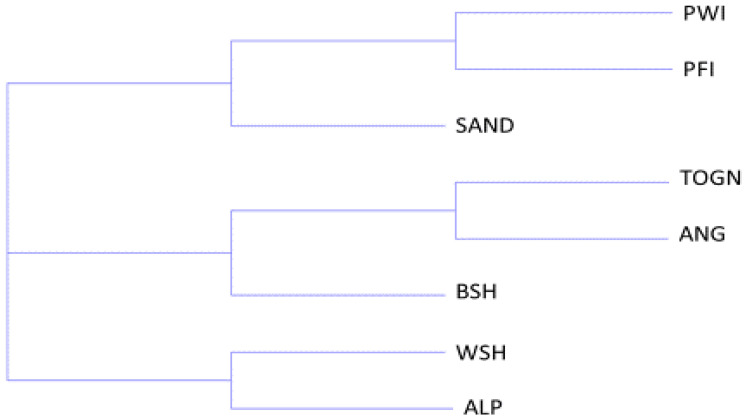
Phylogenetic tree of the eight goat breeds, generated from the pairwise Reynolds’ genetic distance matrix [33]. Abbreviations: WSH—White Shorthaired goat; BSH—Brown Shorthaired goat; ANG—Anglo-Nubian; ALP—Alpine; PFI—Polish Fawn Improved goat; PWI—Polish White Improved goat; SAND—Sandomierska goat; TOGN—Toggenburg goat.

As shown in the neighbor-joining dendrogram (Figure 2), the eight breeds resolved into three main clusters. The first group contained the Polish PWI, PFI, and SAND breeds; the second encompassed the TOGN, ANG, and BSH breeds; and the final cluster grouped the WSH and ALP populations together.

### 3.4. Genetic Structure and Admixture Analysis

**Figure 3:**
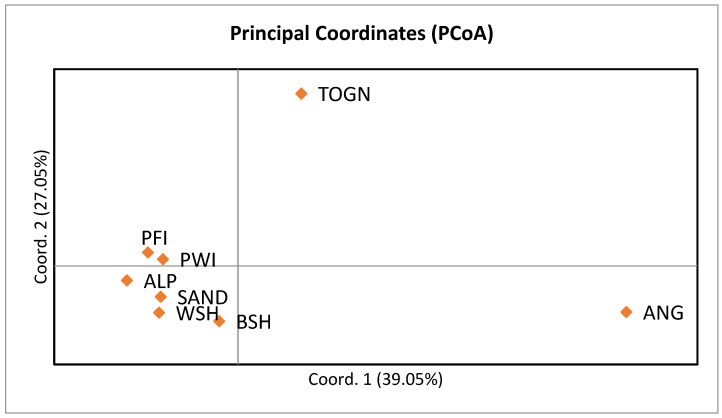
Principal coordinate analysis (PCoA) two-dimensional plot illustrating the relationships among the eight goat breeds. The clustering is based on 15 standardized microsatellite markers analyzed via GenAlEx. Abbreviations: WSH—White Shorthaired goat; BSH—Brown Shorthaired goat; ANG—Anglo-Nubian; ALP—Alpine; PFI—Polish Fawn Improved goat; PWI—Polish White Improved goat; SAND—Sandomierska goat; TOGN—Toggenburg goat.

To further investigate genetic differentiation and ancestral admixture among the eight goat populations, Principal Coordinate Analysis (PCoA) and Bayesian clustering were conducted. As shown in Figure 3, the first two axes of the PCoA accounted for 66.10% of the total genetic variance (39.05% for axis 1 and 27.05% for axis 2). Spatial distribution along these coordinates clearly segregated the cosmopolitan TOGN and ANG breeds from the rest of the study groups. The remaining breeds clustered closely together, exhibiting a moderate to high degree of genetic homogeneity, which is highly consistent with the variation revealed by the pairwise genetic distance matrices and the phylogenetic dendrogram described above (Figure 2).

**Figure 4:**
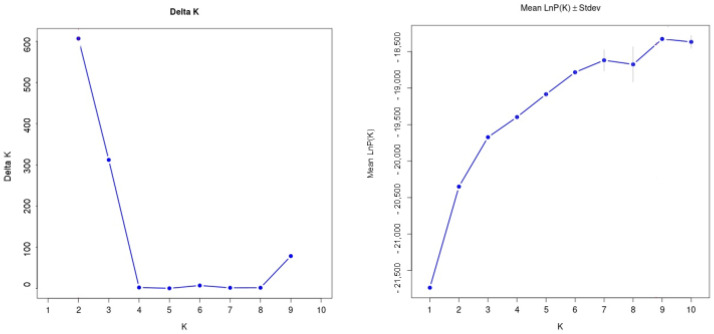
ΔK value inferred with Structure Selector in goat populations using the method by Evanno et al. [36], Raj et al. [37], and Puechmaille statistics [38].

**Figure 5:**
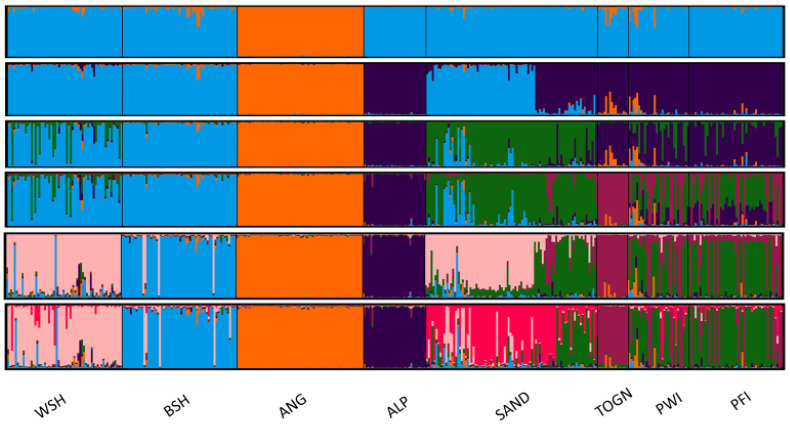
Genetic structure of Czech and Polish goat breeds inferred by Bayesian clustering analysis from K = 2 to K = 8 using STRUCTURE software package [36,37,38,39,40]. Each analyzed individual is represented by a single vertical line divided into colored segments proportional to its archive cohort. Abbreviations: WSH—White Shorthaired goat; BSH—Brown Shorthaired goat; ANG—Anglo-Nubian; ALP—Alpine; PFI—Polish Fawn Improved goat; PWI—Polish White Improved goat; SAND—Sandomierska goat; TOGN—Toggenburg goat.

Bayesian clustering analysis implemented in the STRUCTURE software was utilized to assess fine-scale breed differentiation. According to the Delta K log-likelihood estimation, the most likely number of ancestral populations contributing to the observed genetic variability was K = 6 (Figure 4). To understand the hierarchical structure, clustering results from K = 2 to K = 8 were evaluated (Figure 5). At K = 2, the eight analyzed populations were assigned to two main ancestral groups. Interestingly, except for the highly distinct Anglo-Nubian breed, the other seven breeds shared a high degree of genetic similarity, indicating a common broad ancestral origin.

At (K = 3), the ALP, SAND, TOGN, PWI, and PFI goats began to cluster separately from the other populations. The native Polish Sandomierska (SAND) breed demonstrated a significant level of genetic admixture from almost all studied populations, except for the ANG breed. Similarly, the improved Polish breeds (PWI and PFI) exhibited moderate to high levels of admixture. In contrast, the Czech shorthaired populations (WSH and BSH) maintained lower levels of genetic admixture. Finally, at the optimal (K = 6), six separate and well-defined genetic clusters were identified, demonstrating a population structure that is highly congruent with the phylogenetic dendrogram.

## 4. Discussion

### 4.1. Population Genetic Diversity

Native or local breeds represent historical, cultural, and socio-economic value for each country, as they are characterized by their diversity, resilience, and adaptability to local environmental conditions. For this reason, it is essential to preserve the genetic diversity of native breeds and populations, compared to commercial cosmopolitan breeds, as well as to estimate their level of threat and establish effective management and conservation strategies for the breed, as stated by Negrini et al. [9], Eusebi et al. [17], and Kawecka et al. [23]. Many studies determine the genetic diversity of local breeds and populations using various molecular tools [16,19,41], such as microsatellite markers (beginning in 1990) [7,10,14,21,25], mt DNA [42,43], SNP analysis [13,44], and whole-genome sequencing (WGS) (past decade) [22,45,46,47].

Although the 15 microsatellite markers were polymorphic and highly informative for broad-scale diversity and legacy dataset comparisons, this small panel has inherent limitations. It offers reduced precision for estimating fine-scale admixture, rare alleles, and recent splits and cannot capture genomic regions under selection or adaptive variation. Consequently, our results represent a baseline, population-level assessment of neutral variation. Future research utilizing high-density SNP arrays or whole-genome sequencing will be essential to accurately evaluate genomic diversity, runs of homozygosity (ROH), effective population size (Ne) and selection signatures.

In this work, the results showed that the total number of alleles per locus ranged from four to 22, indicating that the selected microsatellite panels were highly polymorphic and robustly reflected genetic diversity. The effective number of alleles (Ne) per locus ranged from 1.889 to 5.145 (Table 1), representing the substantial genetic variance maintained across the observed breeds. These values were lower than those reported for Chinese goats [48,49], Spanish Guadarrama goat [50], and Algerian and Turkish populations [12]. However, they were comparable to Swiss [7], Italian [9], and Iranian goat breeds [51], as well as the Polish Carpathian goat [24], other Polish autochthonous breeds [23], and Bulgarian breeds [14]. Based on these results, we can conclude that diversity is not merely determined by the number of loci used or the number of breeds monitored but rather by the inherent allelic variants presented within each monitored breed or population. All analyzed markers were highly polymorphic and deemed suitable for biodiversity assessment, with each locus displaying more than four alleles, except for ETH10 (K = 4).

Another crucial parameter for measuring genetic diversity is heterozygosity, particularly the expected heterozygosity (He), which defines the probability that two randomly selected alleles from a population differ [18]. In our dataset, overall expected heterozygosity (He = 0.696) and observed heterozygosity (Ho = 0.684) were well-balanced, indicating a moderate to high level of genetic diversity. This balance further suggests a stable population structure without significant immediate pressure of genetic erosion. From an evolutionary and practical perspective, this is a known phenomenon in genetic conservation. While native Central European breeds still have high genetic diversity, keeping them in small, closed conservation programs limits their population size. This situation, unfortunately, increases local inbreeding. Similar balanced heterozygosity values have been reported for Polish local breeds [25] and Italian goats [9]. Compared to Algerian and Turkish breeds (0.81 and 0.93, respectively) [12] and Iranian breeds (Ho 0.982; He 0.756) [51], our heterozygosity values were slightly lower. This discrepancy shows what happens when populations are split up in Central Europe. Instead of being one large, connected group, traditional breeds live as isolated genetic islands. Heterozygosity deficits in goat populations can generally result from several factors, including inbreeding and genetic drift in small or isolated populations, selection pressure, low mutation rates, or non-random mating, as described by Bensouf et al. [52]. The moderate heterozygosity found in our study shows that we urgently need standardized, local conservation programs. We must carefully plan which animals mate to stop genetic drift, which happens because these breeds are kept isolated to protect them.

When interpreting these indices, sampling constraints must be considered. Inadvertent inclusion of close relatives can underestimate observed heterozygosity and inflate (FIS), while sampling from a few herds may capture local genetic drift rather than breed-wide diversity. Although we avoided related animals when possible, complete pedigree and herd data were not uniformly available. Thus, our results represent the sampled populations rather than exhaustive breed estimates, a limitation particularly relevant for smaller cohorts like Toggenburger (N = 16) and Alpine (N = 32). Furthermore, unequal sample sizes limit this study. Allele counts and private alleles are sensitive to sample size due to the higher probability of detecting rare alleles in larger cohorts. Therefore, the high diversity in Sandomierska goats (N = 88) and the lower counts in Toggenburger (N = 16) must be interpreted cautiously. Because uneven sampling can distort genetic distances and Bayesian clustering, our STRUCTURE results were integrated with FST, PCoA, and breed history rather than used as a standalone metric. Finally, potential genotyping artifacts—such as null alleles, stutter peaks, and large allele dropout—must be acknowledged. For instance, observed deviations from the Hardy–Weinberg equilibrium (Table 1) were interpreted carefully, as they may reflect biological forces (substructure, selection, or non-random mating) or technical scoring errors. The partitioning and loss of genetic variability can be efficiently evaluated using Wright’s F-statistics [53], which include three fixation indices: F_IS_, F_ST_, and F_IT_. The F_IS_ index provides insights into the degree of inbreeding within a subpopulation. In this study, the mean value (F_IS_ = 0.018) was low but positive, indicating negligible inbreeding and only a minor deficit of heterozygotes relative to the Hardy–Weinberg expectations. This pattern may reflect a minor population substructure or localized non-random mating within specific herds, although gene flow among herds could partially mitigate inbreeding effects. The positive F_ST_ value (0.110) demonstrated that only 11% of the total genetic variability can be attributed to differences between breeds, whereas the remaining 89% of genetic variance is due to differences within breeds (incorporating variation among individuals within populations and variation within individuals themselves). A positive value was also recorded for total fixation index F_IT_ (0.125), indicating a 12.5% global deficit of heterozygotes across the entire metapopulation. The F_ST_ and F_IT_ parameters across all loci displayed moderate values. Higher levels of genetic differentiation between breeds have been documented in the three Indian goat breeds [8] and local Albanian goats [54]. On the contrary, lower differentiation values were recorded in native Carpathian goat in Poland [24], in two Bulgarian local goats [14], Chinese goat breeds [48,49], and other Italian breeds [9]. However, our results closely match those observed in Algerian and Turkish breeds [12]. The low (F_IS_) values indicate a higher proportion of heterozygous individuals, which could be explained by high gene flow between breed herds and the avoidance of mating related animals. These positive values may also be a consequence of some loci being homozygous due to natural selection or being linked to other loci affecting morphological, production, or adaptive traits undergoing natural selection, as described by Dixit et al. [8]. From a practical standpoint, the low but positive F_IS_ across our populations suggests that while inbreeding is present due to breed isolation, current breeding strategies remain effective in avoiding severe parental consanguinity.

The total genetic diversity (G_ST_) fully supported the F-statistics, confirming that approximately 10% of the total genetic diversity was distributed among breeds, with the remaining 90% within populations. Keeping 90% of the genetic variation helps these breeds adapt to change. This high diversity acts as a safety net, allowing our national gene resources to survive future climate and environmental shifts. However, lower G_ST_ values were previously found in Chinese breeds (0.0522) [48], Albanian goats [54], and Algerian and Turkish populations (0.054) [12], whereas higher differentiation was reported in Swiss breeds (0.17) [7]. Similar moderated values were also shown in African breeds (0.084) [55]. As stated by Nguluma et al. [55] and Dixit et al. [8], these varying levels of breed differentiation are driven by several factors, including the intensity of historical exchange of breeding material (sires and dams), animal mobility between regions allowing gene flow, genetic drift, and localized selection practices or inbreeding. The mean Shannon’s information index (I) across loci was 1.476, which is consistent with the values reported for Albanian breeds [54] and Iranian goat breeds [51], but lower than those for Bulgarian breeds [14]. Generally, this index reflects the cumulative effectiveness of the genetic markers and underscores a high level of total genetic variability within the studied population, as described by Moravcíkova et al. [56].

### 4.2. Genetic Differentiation Among Breeds

The genetic relationships and microevolutionary dynamics among the studied Czech and Polish goat breeds were comprehensively evaluated using genetic distance matrices, PCoA, and Bayesian admixture analysis. Based on the pairwise Fst matrix, the results indicated a relatively low to moderate level of genetic differentiation, ranging from 0.012 (between the Polish PWI and PFI) to 0.132 (between the ANG and TOGN). Consequently, approximately 1.2–13.2% of the total genetic variance is attributable to breed divergence, whereas the remaining 86.8% to 98.8% resides within within-breed variation, largely driven by high intra-individual allelic diversity. These findings align with previous studies [9,23,54], confirming that genetic differentiation among local or regional (parapatric) breeds is typically lower than that observed between goats of distant geographical or distinct evolutionary origins (such as the Anglo-Nubian goat). Regarding gene flow, the highest number of migrating individuals (Nm) was detected between the Polish PWI and PFI breeds (Nm = 19.935), followed by PWI and SAND (Nm = 16.919), SAND and PFI (Nm = 12.463), and notably between the Czech WSH and Polish SAND populations (Nm = 12.476). The low pairwise Fst values and short genetic distances strongly support high historical gene flow. This pattern persists despite contemporary differences in breeding management, suggesting a shared ancestry and indicating that Central European local breeds mixed freely prior to the establishment of official herd books. This close genetic affinity is predominantly driven by transboundary movement and the exchange of genetic material in the border regions, further reinforced by the historical use of the cosmopolitan Saanen and Alpine goats to enhance milk and cheese production. These results are fully congruent with the phylogenetic and structural analyses, which assign the WSH, BSH, TOGN, PWI, PFI, and SAND breeds to a single evolutionary lineage, distinct from the divergent Anglo-Nubian breed. The Anglo-Nubian goat remains unique due to its African and Asian ancestry, which provides a distinct genomic profile separate from European breeds.

The clustering results obtained from the Bayesian analysis reflect different hierarchical levels of resolution rather than contradictory findings, providing a comprehensive view of the population architecture. At K = 2, the analysis establishes a biologically interpretable baseline, revealing two primary ancestral gene pools and highlighting the macro-structural split between British-origin (Anglo-Nubian) and the remaining Central European groups. This deep split reflects their shared Central European breeding history and a well-documented historical Saanen/Swiss influence, aligning tightly with our PCoA and neighbor-joining dendrogram results, where the Anglo-Nubian consistently emerges as the most divergent breed. At K = 3, a subtle differentiation emerged between the Czech populations (WSH and BSH) and the Polish cohorts. Interestingly, the Sandomierska (SAND) breed did not form an isolated genetic cluster, exhibiting high genetic admixture with both the Polish Improved and the Czech indigenous shorthaired goats. Practically, this lack of distinct genetic boundaries in the SAND goat may represent an evolutionary consequence of past population bottlenecks, where a critically endangered native population was restored using available local genetic material.

Furthermore, in accordance with Bagnicka et al. [26], it is highly probable that Polish populations were historically enhanced through the importation of White Shorthaired goats from the former Czechoslovakia. Specifically, this close genetic relationship directly mirrors documented historical gene flow across the Czech–Polish border: in the late 1980s, local Polish goat populations in Lower Silesia were systematically upgraded using imported Czech WSH bucks, and this genetic material was subsequently disseminated nationwide. The high number of effective migrants identified between SAND–WSH (Nm = 12.476) and PWI–PFI Nm = 19.935) provides clear molecular evidence of this shared agricultural history and ongoing cross-border exchange. This broad genetic affinity suggests that the Sandomierska population historically functioned as an ancestral genetic reservoir or a crossroads for regional transboundary breeds—a phenomenon warranting deeper genomic investigation.

At the statistically optimal (K = 6), as identified by the Evanno (ΔK) method and Puechmaille statistics implemented via STRUCTURE SELECTOR, the defined genetic clusters exhibited a clear microstructure highly congruent with the Reynolds’ distance-based neighbor-joining dendrogram. This confirms that current populations maintain identifiable genetic boundaries despite historical gene flow, capturing the full genomic complexity of the breeds. At this higher resolution, the Anglo-Nubian and Toggenburger separate distinctly as genetically unique assets, which highlights their importance for targeted conservation measures. Crucially, the results revealed high genetic diversity among the populations, with an inbreeding coefficient Fis of 0.018 at the marker level, indicating low levels of inbreeding and substantial intra-population variability. These clustering outcomes demonstrate high consistency with the phylogenetic tree, Fst and PCoA analyses, aligning with findings reported by Manunza et al. [57] for Western European breeds (Alpine goat, Saana goat, Toggenburg), as well as by Kawecka et al. [23,24], Sikora et al. [25] and Bagnicka et al. [26] for Polish breeds, and by Vostry et al. [22] (Czech breeds).

Ultimately, these genetic distance and clustering analyses demonstrate that geographical distribution is a key determinant of population-level genetic diversity, facilitated by frequent gene flow between adjacent populations. This explains why populations within a specific region often remain genetically close despite striking phenotypic divergence, a phenomenon also documented by Kusza et al. [58]. Proximity and shared transboundary history across the Polish–Czech border have clearly superseded official breed boundaries. Consequently, instead of sharp genetic divisions, Central European goats exhibit a smooth, isolation-by-distance clinal variation across regions.

However, this historical introgressive hybridization with Saanen and Alpine goats complicates the conservation of local breeds, as past breeding strategies have compromised their genetic purity. By identifying two broad macro-gene pools alongside six fine-scale clusters, this study establishes a robust molecular framework for the design of genetic conservation programs. These data allow breeders to implement strategies that maximize within-pool diversity while intentionally avoiding excessive, unmonitored admixture between the divergent Alpine/Anglo-Nubian lineages and the core Central European gene pool. Therefore, future conservation programs must utilize these DNA profiles to select breeding bucks and does with minimal outside breed influence, thereby minimizing foreign introgression and restoring the original genetic identity of these indigenous breeds. To build upon this 15-marker baseline assessment, future research should integrate high-density SNP arrays or whole-genome sequencing. Combining genomic datasets with complete pedigree records and sex-specific marker panels (such as Y-chromosome haplotypes and mtDNA sequencing) will be crucial to reconstruct sex-biased gene flow and provide highly precise, genomic-based inbreeding estimates.

## 5. Conclusions

An analysis of 15 microsatellite markers revealed substantial genetic diversity among the eight goat breeds. Although five loci (SRCRSP23, MAF65, TGLA53, INRA172, CSRD247) deviated significantly from HWE due to potential technical artifacts or biological factors, the majority (10 loci) conformed to HWE expectations. This demonstrates that the marker panel is robust and highly suitable for population genetic inference. Diversity by principal coordinate analysis (PCoA) and population structure revealed two main gene pools; therefore, the geographical origin of the breeds should be taken into consideration while deciding conservation and improvement options for these breeds. These findings have practical implications for conservation and breeding efforts, guiding targeted breeding strategies to preserve and enhance desirable traits within each breed. Ultimately, preserving the genetic variability of local breeds is crucial for each country. It helps animals adapt to local climatic conditions and integrate them into the rural economy, as maintaining a high level of genetic diversity, especially in indigenous breeds, supports global conservation strategies, is a source of economic development and promotes agritourism.

## Figures and Tables

**Table 1:** Parameters of genetic diversity over the population for each microsatellite marker.

Locus	K	Na	Ne	Ho	He	Ht	F(null)	PIC	HWe
SRCRSP23	15	8.875	5.145	0.704	0.714	0.880	0.006	0.862	***
MAF65	23	11.250	4.933	0.801	0.774	0.861	−0.015	0.844	***
MCM527	8	6.125	3.959	0.718	0.736	0.788	0.010	0.744	ns
BM1329	9	6.125	3.237	0.661	0.666	0.731	0.003	0.672	ns
ETH10	4	3.000	1.889	0.473	0.465	0.542	−0.006	0.461	ns
ILSTS11	9	5.500	3.388	0.667	0.660	0.769	−0.004	0.741	ns
TGLA53	11	5.750	2.555	0.543	0.599	0.660	0.035	0.621	***
SPS113	16	7.750	4.705	0.747	0.771	0.841	0.013	0.820	ns
SRCSP5	15	8.125	4.318	0.763	0.738	0.834	−0.014	0.799	***
SRCSP8	13	7.750	3.948	0.656	0.712	0.812	0.033	0.796	ns
INRA172	14	7.125	4.385	0.730	0.767	0.838	0.020	0.817	***
INRA006	12	8.000	5.128	0.810	0.752	0.838	−0.033	0.835	ns
INRA63	7	5.000	2.726	0.589	0.617	0.691	0.017	0.628	ns
INRA23	12	7.000	3.599	0.710	0.720	0.746	0.005	0.719	ns
CSRD247	23	9.250	4.704	0.690	0.755	0.912	0.037	0.804	ns
**Mean**	12.733	7.108	3.908	0.684	0.696	0.783	0.007	0.744	0.018

Note: K—number of identified alleles, Na—number of alleles, Ne—number of effective alleles, Ho—observed heterozygosity, He—expected heterozygosity, Ht—total expected heterozygosity, F(null)—null allele frequency, PIC—polymorphic information content, HWe—Hardy–Weinberg equilibrium, ns = not significant. *** *p* ˂0.001.

**Table 2:** Wright’s F-statistic analysis.

Locus	F_IS_	F_IT_	F_ST_	I	G_ST_	G_IS_	Nm
SRCRSP23	0.014	0.200	0.189	1.685	0.179	0.027	1.074
MAF65	−0.034	0.069	0.100	1.838	0.090	−0.021	2.240
MCM527	0.024	0.089	0.066	1.504	0.055	0.037	3.547
BM1329	0.007	0.096	0.090	1.322	0.079	0.020	2.526
ETH10	−0.018	0.127	0.143	0.783	0.133	−0.005	1.503
ILSTS11	−0.009	0.133	0.141	1.289	0.131	0.004	1.524
TGLA53	0.093	0.177	0.092	1.159	0.081	0.107	2.457
SPS113	0.031	0.112	0.084	1.684	0.073	0.044	2.721
SRCSP5	−0.034	0.085	0.116	1.615	0.106	−0.021	1.908
SRCSP8	0.079	0.192	0.123	1.561	0.111	0.092	1.790
INRA172	0.048	0.129	0.085	1.630	0.074	0.061	2.679
INRA006	−0.078	0.033	0.103	1.693	0.093	−0.065	2.184
INRA63	0.045	0.147	0.107	1.193	0.096	0.058	2.093
INRA23	0.013	0.048	0.035	1.482	0.024	0.026	6.843
CSRD247	0.086	0.243	0.172	1.705	0.161	0.099	1.201
**Mean**	0.018	0.125	0.110	1.476	0.100	0.031	2.419

Note: Fixation index (F_IS_, F_IT_, F_ST_), I—Shannon’s information index, G_ST_—genetic diversity among population at each locus, G_IS_—inbreeding coefficient within individuals for each locus, Nm = number of migrants; Nm = [(1/F_ST_) − 1]/4.

**Table 3:** Parameters of genetic diversity over loci for each goat population.

Breed	N	Na	Ne	I	Ho	He	F	PA
**WSH**	126	8.400	4.120	1.594	0.697	0.721	0.028	3
**BSH**	110	7.333	3.712	1.489	0.699	0.704	0.002	3
**ANG**	79	5.267	2.663	1.100	0.599	0.576	−0.041	3
**ALP**	81	5.400	3.883	1.435	0.702	0.725	0.041	2
**SAND**	151	10.067	4.712	1.713	0.686	0.744	0.073	11
**TOGN**	80	5.333	3.180	1.269	0.654	0.629	−0.038	4
**PWI**	111	7.400	4.478	1.601	0.715	0.735	0.029	2
**PFI**	115	7.667	4.515	1.608	0.721	0.735	0.021	5

Note: N—number of typed alleles; Na—mean number of different alleles; Ne—number of effective alleles; I—Shannon’s information index; Ho—observed heterozygosity; He—expected heterozygosity; F—inbreeding coefficient; PA—number of private alleles. Abbreviations: WSH—White Shorthaired goat; BSH—Brown Shorthaired goat; ANG—Anglo-Nubian; ALP—Alpine; PFI—Polish Fawn Improved goat; PWI—Polish White Improved goat; SAND—Sandomierska goat; TOGN—Toggenburg.

**Table 4:** Summary results of AMOVA analysis and F-statistics, revealing the distribution of genetic diversity.

Source	df	SS	MS	Var.	%	F-Statistics	Value
Among Population	6	377.008	62.835	0.615	10%	F_ST_	0.109
Among Individuals Within Populations	390	2139.537	5.486	0.185	3%	F_IS_	0.035
Within Individuals	398	2036.400	5.117	5.117	86%	F_IT_	0.140
Among Regions	1	90,386	90,386	0.030	1%		
**Total**	795	4643.330		5.946	100%	---	---

Note: df—degree of freedom, SS—sum of squares, MS—mean square, Var.—estimated variation, %—percentage of variation, F_IS_—fixation indices (among populations); F_ST_—fixation indices (among individuals within populations); F_IT_—fixation indices (within individuals).

**Table 5:** Pairwise population differentiation (F_ST_) value (below diagonal) and gene flow (Nm) estimated (above diagonal) among the eight studied goat breeds.

Breed	WSH	BSH	ANG	ALP	SAND	TOGN	PWI	PFI
**WSH**	0.000	8.241	1.944	5.487	12.476	2.197	8.010	6.898
**BSH**	0.029	0.000	2.254	3.948	8.334	2.174	6.822	4.863
**ANG**	0.114	0.100	0.000	1.657	2.067	1.649	2.165	1.929
**ALP**	0.044	0.060	0.131	0.000	5.631	2.079	6.009	6.037
**SAND**	0.020	0.029	0.108	0.043	0.000	2.440	16.919	12.463
**TOGN**	0.102	0.103	0.132	0.107	0.093	0.000	3.055	2.782
**PWI**	0.030	0.035	0.104	0.040	0.015	0.076	0.000	19.935
**PFI**	0.035	0.049	0.115	0.040	0.020	0.082	0.012	0.000

Abbreviations: WSH—White Shorthaired goat; BSH—Brown Shorthaired goat; ANG—Anglo-Nubian; ALP—Alpine; PFI—Polish Fawn Improved goat; PWI—Polish White Improved goat; SAND—Sandomierska goat; TOGN—Toggenburg goat.

**Table 6:** Pairwise population matrix of Nei (DS) genetic distance [30].

Breed	WSH	BSH	ANG	ALP	SAND	TOGN	PWI	PFI
**WSH**	0.000							
**BSH**	0.164	0.000						
**ANG**	0.609	0.491	0.000					
**ALP**	0.283	0.398	0.788	0.000				
**SAND**	0.117	0.176	0.594	0.291	0.000			
**TOGN**	0.605	0.603	0.611	0.668	0.560	0.000		
**PWI**	0.197	0.222	0.552	0.264	0.095	0.396	0.000	
**PFI**	0.232	0.322	0.651	0.258	0.130	0.439	0.076	0.000

Abbreviations: WSH—White Shorthaired goat; BSH—Brown Shorthaired goat; ANG—Anglo-Nubian; ALP—Alpine; PFI—Polish Fawn Improved goat; PWI—Polish White Improved goat; SAND—Sandomierska goat; TOGN—Toggenburg goat.

**Table 7:** Genetic distance between goat populations, Rogers’ genetic distance (DR) [32], is below the diagonal, and the distance according to Reynolds et al. [33] is above the diagonal.

Breed	ALP	ANG	BSH	PFI	PWI	SAND	TOGN	WSH
**ALP**	0	0.223	0.104	0.077	0.076	0.078	0.192	0.069
**ANG**	0.408	0	0.183	0.200	0.179	0.196	0.224	0.202
**BSH**	0.269	0.344	0	0.086	0.059	0.059	0.179	0.057
**PFI**	0.237	0.382	0.249	0	0.026	0.034	0.145	0.056
**PWI**	0.229	0.357	0.200	0.130	0	0.025	0.136	0.046
**SAND**	0.231	0.381	0.201	0.151	0.125	0	0.166	0.037
**TOGN**	0.384	0.404	0.364	0.310	0.301	0.336	0	0.172
**WSH**	0.222	0.382	0.190	0.194	0.169	0.161	0.346	0

Abbreviations: WSH—White Shorthaired goat; BSH—Brown Shorthaired goat; ANG—Anglo-Nubian; ALP—Alpine; PFI—Polish Fawn Improved goat; PWI—Polish White Improved goat; SAND—Sandomierska goat; TOGN—Toggenburg goat.

## Data Availability

The data presented in the present study are available in the article and Supplementary Material.

## Supplementary Materials

The following supporting information can be downloaded at: https://www.mdpi.com/article/10.3390/ani16111660/s1, Table S1: Characteristics of 15 microsatellite loci, including in the multiplex PCR reaction, Dey, product size (bp), Annealing temperature (°C); Table S2: Number of identified alleles per locus (Na), effective number of alleles per locus (Ne), Shannon’s information index (I), heterozygosity: observed (Ho) and expected (He), inbreeding coefficient (F), Chi-Square tests for Hardy–Weinberg equilibrium (HWE), ns = not significant, * *p* < 0.05, ** *p* < 0.01, *** *p* < 0.001, in all studied microsatellite markers by breed.
