# Supplementary material for: Genetic Diversity and Population Structure Among Czech and Polish Goat Breeds Assessed Using Microsatellite Markers
Source: Animals (Basel). 2026 May 29;16(11):1660. doi: 10.3390/ani16111660 (PMC13255871; doi:10.3390/ani16111660)
Supplement: Supplementary file 1 [file animals-16-01660-s001.zip › animals-4331294-supplementary.pdf]

Supplementary

Table S1. Characteristics of 15 microsatellite loci including in the multiplex PCR reaction, Dey, product size (bp), Annealing temperature (°C)

Microsatellite markers	Chr. No	Primer sequences 5´- 3´	Label	Product size (bp)	Annealing Temperature (°C)
**Multiplex1**					
INRA06	BTA3	F: AGGAATATCTGTATCAACCGCAGTC<br>R: CTGAGCTGGGGTGGGAGCTATAAATA	FAM	100-130	49.5
INRA063 (D1855)	18	F: ATTTGCACAAGCTAAATCTAACC<br>R: AAACCACAGAAATGCTTGGAAG	VIC	160-190	49.5
INRA23	3	F: GTAGAGCTACAAGATAAACTTC<br>R: TAACTACAGGGTGTTAGATGAACT	FAM	190-220	49.5
CSRD247	14	F: GGACTTGCCAGAACTCTGCAAT<br>R: CACTGTGGTTTGTATTAGTCAGG	PET	220-250	49.5
**Multiplex2**					
SRCRSP23	UNK	F: TGAACGGGTAAAGATGTG<br>R: TGTTTTTAATGGCTGAGTAG	FAM	80-120	55
MAF65	OAR15	F: AAAGGCCAGAGTATGCAATTAGGAG<br>R: CCACTCCTCCTGAGAATATAACATG	VIC	115-160	55
MCM527	5	F: GTCCATTGCCTCAAATCAATTC<br>R: AAACCACTTGACTACTCCCCAA	PET	160-190	55
BM1329	6	F: TTGTTTAGGCAAGTCCAAAGTC<br>R: AACACCGCAGCTTCATCC	FAM	160-180	55
ETH10	15	F: GTTCAGGACTGGCCCTGCTAACA<br>R: CCTCCAGCCCACTTTCTCTTCTC	VIC	200-230	55
ILSTS11	BTA14	F: GCTTGCTACATGGAAAGTGC<br>R: CTAAAATGCAGAGCCCTACC	FAM	250-300	55
**Multiplex3**					
TGLA53 (D1653)	16	F: GCTTTCAGAAATAGTTTGCATTCA<br>R: ATCTTCACATGATATTACAGCAGA	VIC	130-190	55
SPS113	BTA10	F: CCTCCACACAGGCTTCTCTGACTT<br>R: CCTAACTTGCTTGAGTTATTGCCC	PET	130-160	55
SRCRSP5	18	F: GGACTCTACCAACTGAGCTACAAG<br>R: TGAAATGAAGCTAAAGCAATGC	FAM	120-180	55
SRCRSP8	UNK	F: TGCGGTCTGGTTCTGATTTCAC<br>R: CCTGCATGAGAAAGTCGATGCTTAG	FAM	210-260	55
INRABERN172	BTA26	F: CCACTTCCCTGTATCCTCCT<br>R: GGTGCTCCCATTGTGTAGAC	VIC	230-260	55

Table S2. Number of identified alleles per locus (Na), effective number of alleles per locus (Ne), Shannon's information index (I), heterozygosity: observed (Ho) and expected (He), inbreeding coefficient (F), Chi-Square Tests for Hardy-Weinberg Equilibrium (HWE), ns=not significant, * P<0.05, ** P<0.01, *** P<0.001, in all studied microsatellite markers by breed

Pop		SRCRSP23	MAF65	MCM527	BM1329	ETH10	ILSTS11	TGLA 53	SPS113	SRCSP5	SRCSP8	INRA172	INRA006	INRA 63	INRA 23	CSRD247
WSH	Na	13	13	6	7	3	7	6	9	8	9	7	9	7	11	11
	Ne	7.075	7.305	3.715	3.009	1.699	3.293	3.062	4.5	2.725	3.075	5.038	5.846	3.259	3.802	4.401
	I	2.233	2.18	1.451	1.354	0.743	1.41	1.328	1.771	1.367	1.512	1.754	1.866	1.463	1.662	1.822
	Ho	0.746	0.898	0.644	0.678	0.475	0.644	0.627	0.678	0.695	0.576	0.746	0.915	0.593	0.78	0.763
	He	0.859	0.863	0.731	0.668	0.411	0.696	0.673	0.778	0.633	0.675	0.801	0.829	0.693	0.737	0.773
	HWE	***	ns	ns	ns	ns	ns	***	ns	ns	ns	ns	ns	**	ns	ns
	F	0.131	-0.041	0.119	-0.015	-0.154	0.075	0.069	0.128	-0.098	0.146	0.07	-0.104	0.144	-0.058	0.013
BSH	Na	10	10	7	7	3	5	5	7	8	8	6	9	6	8	11
	Ne	3.992	3.445	4.09	2.264	2.174	3.101	3.114	2.771	5.18	2.722	4.58	5.945	2.805	3.554	5.945
	I	1.72	1.599	1.553	1.119	0.908	1.28	1.236	1.321	1.776	1.36	1.639	1.895	1.33	1.572	2.025
	Ho	0.644	0.712	0.847	0.475	0.627	0.729	0.78	0.661	0.763	0.644	0.763	0.814	0.661	0.661	0.712
	He	0.749	0.71	0.756	0.558	0.54	0.678	0.679	0.639	0.807	0.633	0.782	0.832	0.643	0.719	0.832
	HWE	***	ns	**	ns	ns	ns	ns	ns	ns	ns	ns	ns	**	***	***
	F	0.141	-0.003	-0.122	0.15	-0.161	-0.076	-0.149	-0.034	0.055	-0.018	0.024	0.022	-0.027	0.08	0.144
ANG	Na	2	10	6	3	3	3	4	5	6	7	6	7	4	6	7
	Ne	1.031	3	2.683	1.862	2.24	1.933	1.931	3.431	2.084	2.222	3.208	4.005	2.969	3.912	3.441
	I	0.079	1.574	1.187	0.708	0.888	0.708	0.797	1.387	0.979	1.143	1.359	1.567	1.164	1.554	1.407
	Ho	0.031	0.692	0.708	0.354	0.538	0.662	0.538	0.785	0.569	0.554	0.692	0.846	0.662	0.677	0.677
	He	0.03	0.667	0.627	0.463	0.553	0.483	0.482	0.709	0.52	0.55	0.688	0.75	0.663	0.744	0.709
	HWE	ns	ns	ns	ns	ns	*	ns	ns	ns	ns	ns	ns	ns	ns	ns
	F	-0.016	-0.039	-0.128	0.235	0.027	-0.37	-0.117	-0.107	-0.094	-0.007	-0.006	-0.128	0.002	0.091	0.046

ALP	Na	6	9	5	5	2	5	4	5	6	6	6	5	5	5	7
	Ne	4.231	4.633	3.378	4.039	1.789	4.267	2.809	3.886	4.258	4.719	4.511	2.989	3.883	4.055	4.8
	I	1.551	1.755	1.401	1.456	0.633	1.511	1.113	1.416	1.527	1.624	1.619	1.291	1.451	1.474	1.706
	Ho	0.938	0.875	0.452	0.844	0.344	0.719	0.5	0.719	0.906	0.688	0.781	0.677	0.71	0.742	0.633
	He	0.764	0.784	0.704	0.752	0.441	0.766	0.644	0.743	0.765	0.788	0.778	0.665	0.742	0.753	0.792
	HWE	ns	ns	***	ns	ns	ns	ns	***	ns	ns	ns	**	ns	ns	ns
	F	-0.228	-0.116	0.358	-0.121	0.22	0.061	0.224	0.032	-0.184	0.128	-0.004	-0.018	0.044	0.015	0.2
SAND	Na	12	15	7	8	4	8	9	14	11	10	11	11	5	9	17
	Ne	6.56	5.49	3.607	3.49	1.889	4.912	2.493	6.945	3.707	5.488	5.325	7.295	2.056	3.781	7.648
	I	2.12	1.997	1.53	1.524	0.818	1.727	1.293	2.145	1.692	1.943	1.878	2.115	0.997	1.615	2.304
	Ho	0.693	0.795	0.784	0.659	0.545	0.694	0.534	0.943	0.625	0.739	0.701	0.886	0.443	0.614	0.636
	He	0.848	0.818	0.723	0.713	0.471	0.796	0.599	0.856	0.73	0.818	0.812	0.863	0.514	0.736	0.869
	HWE	***	***	ns	ns	ns	***	***	**	ns	ns	*	ns	ns	ns	***
	F	0.182	0.027	-0.085	0.076	-0.159	0.128	0.108	-0.102	0.144	0.097	0.137	-0.027	0.137	0.166	0.268
TOGN	Na	6	7	6	4	3	4	6	9	7	5	6	5	3	4	5
	Ne	3.879	3.141	5.389	2.994	1.784	1.571	2.498	5.224	5.224	3.18	3.879	1.6	2.169	3.22	1.947
	I	1.53	1.448	1.735	1.197	0.773	0.7	1.252	1.889	1.745	1.315	1.518	0.805	0.921	1.241	0.961
	Ho	0.813	0.75	0.938	0.813	0.313	0.438	0.563	0.625	0.938	0.688	0.75	0.438	0.375	0.813	0.563
	He	0.742	0.682	0.814	0.666	0.439	0.363	0.6	0.809	0.809	0.686	0.742	0.375	0.539	0.689	0.486
	HWE	ns	ns	*	ns	ns	ns	**	ns	ns	ns	**	ns	ns	ns	ns
	F	-0.095	-0.1	-0.151	-0.22	0.289	-0.204	0.062	0.227	-0.159	-0.003	-0.011	-0.167	0.304	-0.178	-0.157
PWI	Na	11	12	6	7	3	5	6	7	9	8	8	9	5	6	9
	Ne	7.792	7.119	3.725	3.527	1.909	3.883	2.356	5.429	6.628	3.247	4.598	6.385	2.418	3.13	5.018
	I	2.176	2.166	1.482	1.495	0.81	1.436	1.154	1.804	2.025	1.571	1.717	1.95	1.101	1.317	1.805
	Ho	0.933	0.935	0.645	0.613	0.548	0.677	0.345	0.774	0.839	0.548	0.677	0.968	0.71	0.71	0.806
	He	0.872	0.86	0.732	0.716	0.476	0.742	0.576	0.816	0.849	0.692	0.783	0.843	0.586	0.681	0.801
	HWE	**	ns	ns	ns	ns	ns	***	ns	ns	*	ns	ns	ns	ns	ns
	F	-0.071	-0.088	0.118	0.145	-0.152	0.088	0.401	0.051	0.012	0.208	0.134	-0.147	-0.21	-0.043	-0.007
PFI	Na	11	14	6	8	3	7	6	6	10	9	7	9	5	7	7
	Ne	6.602	5.333	5.086	4.712	1.63	4.148	2.175	5.453	4.741	6.929	3.945	6.961	2.245	3.334	4.435

I	2.067	1.988	1.692	1.725	0.688	1.543	1.099	1.738	1.808	2.017	1.554	2.051	1.117	1.42	1.612
Ho	0.833	0.75	0.729	0.854	0.396	0.771	0.458	0.792	0.771	0.813	0.729	0.938	0.563	0.688	0.729
He	0.849	0.813	0.803	0.788	0.387	0.759	0.54	0.817	0.789	0.856	0.747	0.856	0.554	0.7	0.775
HWE	***	***	ns	ns	ns	ns	***	ns	***	ns	***	ns	ns	ns	ns
F	0.018	0.077	0.092	-0.084	-0.024	-0.016	0.151	0.031	0.023	0.05	0.023	-0.095	-0.014	0.018	0.059

Abbreviations: Czech populations: WSH - White Shorthaired goat, BSH - Brown Shorthaired goat, ANG - Anglo-Nubian, ALP - Alpine; Polish populations: SAND - Sandomierska goat, TOGN - Toggenburger goat; PWI - Polish White Improved goat, PFI - Polish Fawn Improved goat
